# A Novel Entry/Uncoating Assay Reveals the Presence of at Least Two Species of Viral Capsids During Synchronized HIV-1 Infection

**DOI:** 10.1371/journal.ppat.1005897

**Published:** 2016-09-30

**Authors:** Claire Da Silva Santos, Kevin Tartour, Andrea Cimarelli

**Affiliations:** 1 CIRI, Centre International de Recherche en Infectiologie, 46 Allée d’Italie, Lyon F69364, France; 2 INSERM, U1111, 46 Allée d’Italie, Lyon, F69364, France; 3 Université Claude Bernard Lyon I, 46 Allée d’Italie, Lyon, F69364, France; 4 CNRS, UMR5308, 46 Allée d’Italie, Lyon, F69364, France; 5 Ecole Normale Supérieure de Lyon, 46 Allée d’Italie, Lyon, F69364, France; 6 Université de Lyon, Lyon, France; Fred Hutchinson Cancer Research Center, UNITED STATES

## Abstract

To better characterize the behavior of HIV-1 capsids we developed EURT, for Entry/Uncoating assay based on core-packaged RNA availability and Translation. EURT is an alternative to Blam-Vpr, but as reporter RNA translation relies on core opening, it can be used to study viral capsids behavior. Our study reveals the existence of two major capsid species, a dead end one in which the viral genome is readily exposed to the cytoplasm and a functional one in which such exposure requires artificial core destabilization. Although reverse transcription drives a faster loss of susceptibility of viral cores to high doses of PF74, it does not lead to higher exposure of the viral genome, implying that viral cores protect the genome irrespectively of reverse transcription. Lastly, IFNα drifts cores from functional to non-functional species, revealing a novel core-destabilizing activity. This assay sheds new light on the behavior of viral cores inside target cells.

## Author Summary

Following viral-to-cellular membrane fusion, the HIV-1 genome is propelled inside the cell as part of an higher order nucleoproteic structure often referred to as viral core, or capsid. Here, we have developed a novel entry/uncoating assay based on the degree of exposure of a virion-packaged mRNA reporter to the translation machinery (EURT). Using this assay, we highlight here that at least two measurable kinds of viral capsids coexist during HIV-1 infection: one defined as open, in which the viral genome is readily accessible to translation and another that we define as closed, in which access to the genome is prevented until the artificial destabilization of capsids. Our data points to the former as dead-end products of infection and indicate the latter as the commonly referred infectious viral cores. Interestingly, we show here that despite the fact that reverse transcription reshapes viral cores, these structures maintain an exquisite ability to shield the viral genome from the cytoplasmic environment. Finally, IFNα that negatively impacts HIV-1 replication increases the proportion of open viral cores to the detriment of closed ones, suggesting a core-destabilizing activity driven by interferon-regulated proteins. Overall, this assay sheds new light on the behavior of viral cores inside target cells.

## Introduction

The correct entry and migration of viral genomes, as well as their protection from assaults driven by cellular effectors represent key steps for the successful infection of target cells. In the case of the HIV-1 lentivirus, the binding of the viral envelope protein gp120 to its cellular receptor CD4 and its co-receptors CCR5 or CXCR4 triggers the fusion between the viral and the cell membranes. This event leads to the cytoplasmic release of viral nucleoprotein complexes that contain the viral genome, as well as viral and cellular proteins (VNCs, more often referred to as cores, or capsids).

Viral cores are higher order structures formed by the Capsid protein (CA) and display a clearly identifiable conical-shape form inside HIV-1 virion particles [1, 2]. A number of studies have now solidly established that CA participates not only to the docking of VNCs to the nuclear pore, but also to post-nuclear entry events, implying that this protein must remain somehow associated to the viral genome beyond its entry into the nucleus [3–11]. However, so far only one study reported intact cores in cells infected with WT HIV-1 [12], while a second failed to visualize them unless hyperstable capsid mutants were used [13]. This is in contrast with the relative straightforward visualization by electron microscopy of intact viral capsids juxtaposed to nuclear pores for a number of other viruses, such as HCMV, HBV, HSV and adenoviruses [14–17] comprehensively reviewed in [18].

This paucity of documentation in the literature for HIV-1 may simply reflect the fact that the vast majority of HIV-1 VNCs are rapidly restructured in the cell cytoplasm, losing their recognizable cone-shaped appearance and shedding most, albeit not all, of their CA protein. This can be due to several causes such as an intrinsic instability of viral cores [19, 20]; the destabilizing action of cellular factors [21–26], or even the process of reverse transcription itself, that is likely to represent a drastic biochemical change within cores [5, 12, 27–30].

The restructuration of viral cores is technically challenging to study and it is for the moment unclear whether one or multiple conformations of VNCs are present during infection.

To complexify the issue, a large proportion of HIV-1 viral particles entering target cells fail to yield an infectious provirus [31–34], indicating that both functional and non-functional VNCs species are present during infection. At this point, it is entirely unappreciated whether this major phenotypic difference is intrinsic to viral particles, perhaps linked to an inefficient mode of virion assembly, or extrinsic, i.e. determined by a cellular environment that manages -somehow- to inactivate a large proportion of incoming viruses. It is interesting to note that a great heterogeneity in the shape of viral cores isolated directly from virion particles has been reported [1, 35], although the functional relevance of such morphological differences could not be explored further. These results support the notion that viral capsids may not be -and may not behave as- a single homogeneous entity.

Most of the assays in use to study HIV-1 viral-to-cellular membranes fusion and uncoating rely on the cytoplasmic liberation of virion-incorporated proteins (for example Blam-Vpr, [36], core purification from cell lysates [37, 38], qPCR of reverse transcription intermediates, or in the case of imaging also on fluorescent lipids, proteins or viral genome labeling [3, 5, 7, 30, 39–41]. These assays provide important data, but by their nature reveal only indirect information about the functionality of viral nucleoprotein complexes and little to no knowledge about how these structural changes may be perceived by the cellular environment. We felt that our comprehension of these phases could benefit from the development of a novel entry assay that could relate about the status of viral genomic RNA in target cells.

To this end, we have developed a novel entry/uncoating assay for HIV-1 that is based on the direct translation of a viral genomic RNA mimic coding a *luciferase* reporter that is delivered in target cells after viral to cellular membrane fusion in viral cores. The results that we have obtained indicate that in its simplest usage, this assay represents an useful alternative to the widely used Blam-Vpr entry test [36], likely more amenable to use in large screens for HIV entry inhibitors thanks to its luciferase-based readout. Most importantly, we show here that with a slightly more complex experimental setup, notably through the combined use of the capsid-binding compound PF74 [42, 43], this assay can reveal important information on the behavior of viral cores during the early phases of infection.

The result we have obtained indicate for the first time that two clearly measurable species of viral cores co-exist during synchronized HIV-1 infection of target cells: one in which the access of the translation machinery to the viral genome is prevented until the destabilization of fullerene cones with high doses of PF74 and another in which instead the viral genome is constitutively exposed to translation. Time of addition experiments of PF74, as well as the use of a modified EURT assay in which the reporter RNA is co-packaged in reverse transcription-competent viral particles, indicate that the first species reflect the behavior of what can be defined as functional viral cores, while the second represent dead end products of infection. We determine here that reverse transcription drives structural changes that can be measured as a more rapid loss in the susceptibility of viral cores to high doses of PF74. However, the EURT assay reveals also that viral nucleoprotein complexes that have undergone major structural changes during reverse transcription are still able to protect the viral genome from the cellular environment. Finally, we show here that while the ratio between these two species of viral capsids does not vary substantially among different cell types, it can be shifted upon IFNα-stimulation of IFN-sensitive target cells, such as monocyte-derived dendritic cells (MDDCs), highlighting the existence of viral core destabilizing, or degradative, activities that are specified by IFN effectors.

## Results

### Presentation of the Entry/Uncoating assay based on core-packaged RNA availability and Translation (EURT), a novel HIV-1 entry/uncoating assay based on the direct translation of a viral genomic RNA mimic

The present study had two purposes: first, the development of a novel entry assay for HIV-1 that could be more amenable to use than currently available ones; second, the attempt to provide novel insights into the fate/s of the viral genome and of the viral core that contains it.

The HIV-1 RNA genome is incorporated into virion particles thanks to a specific interaction between the packaging sequence (Ψ) and the nucleocapsid domain of the Gag polyprotein [44, 45]. Upon virion maturation and Gag processing, this genome is contained within the viral core.

**Fig 1:**
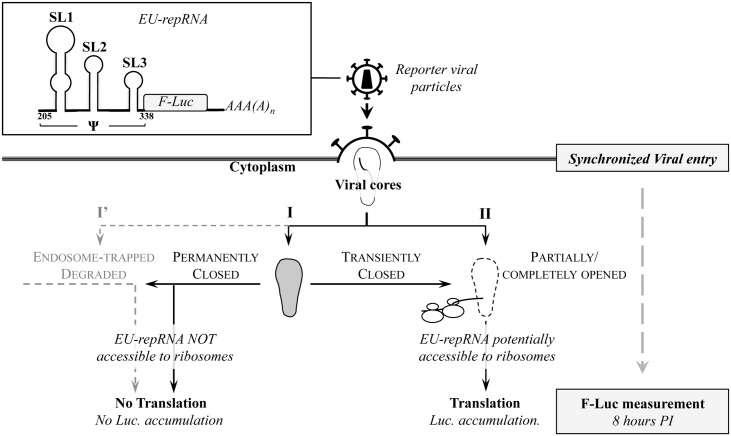
Presentation of the Entry/Uncoating assay based on core-packaged RNA availability and Translation (EURT). An entry/uncoating reporter mRNA coding Luciferase (EU-repRNA) was engineered by appending a minimal HIV-1 packaging sequence (Ψ) upstream of the *Firefly luciferase (F-Luc*, followed by a polyA signal). By virtue of its Ψ sequence, this mRNA behaves as genomic viral RNA and is therefore encapsidated into virion particles and then protected inside viral cores. When HIV-1 virions bearing this reporter are used to challenge target cells, the availability of EU-repRNA to the cytoplasmic translation machinery will be in large measure influenced by the fate and status of the viral core. The cartoon depicts the possible fates of viral cores upon entry, according to data published in the field concerning the early phases of the life cycle of HIV. SL: stem loop; numbers refer to the HIV-1 sequence #K02013. EU-repRNA expression is obtained upon DNA transfection in virus-producing cells and is driven by a CMV promoter (the phase of virion production is not detailed for simplicity).

To engineer a reporter viral genomic RNA, a minimal packaging sequence derived from HIV-1 was inserted upstream of the *Firefly luciferase* open reading frame (*F-Luc*, followed by a polyA signal), to yield an otherwise prototypical cellular mRNA (named hereafter EU-repRNA for entry/uncoating reporterRNA, Fig 1). To isolate viral entry from other steps of the retroviral life cycle and notably reverse transcription, this mRNA lacks other HIV-derived sequences. Indeed, the inherent RNase activity associated to the process of reverse transcription would have likely altered the levels of viral RNA (vRNA), complexifying the analysis of the results of this assay.

HIV-1 reporter viral particles are produced by transient transfection of HEK293T cells with DNAs coding the structural proteins Gag-Pro-Pol, the HIV-1 Env and EU-repRNA. Viral particles are then purified by ultracentrifugation through a 25% sucrose cushion (w/v) and quantified by exogenous-RT (exo-RT) or p24 ELISA against standards of known infectious titers. Purified virions are used to challenge target cells in a synchronized infection (2 hours at 4°C, prior to cell washing and induction of viral entry by shifting the temperature at 37°C) and cells are then lysed for luciferase activity measurement. Based on pilot experiments, a fixed time of eight hours post infection was chosen for most of the experiments.

As schematically depicted in Fig 1, EU-repRNA is delivered to target cells after viral-to-cellular membrane fusion within viral cores. Viral nucleoprotein complexes that are directly degraded, or trapped in endosomal vesicles will not be accessible to the translational machinery (I’), in contrast to those released in the cell cytoplasm. In this latter case however, the exposure of the viral genome to ribosomes and to all the initiation factors required for translation will depend on the conformation of viral cores. Viral cores may remain permanently closed (I, defined here as maintaining their well-ordered fullerene cone structure, [46, 47], can be from the start in an opened or partially opened conformation in which the viral RNA can be accessible to the translation machinery (II, defined here as having lost their highly ordered conformation in contraposition to I), or can shift from a closed to an open state over time. Albeit oversimplified, this view encompasses the two opposite models of HIV-1 uncoating according to which VNCs remain either closed to allow the termination of reverse transcription [12, 48], or -on the contrary- open to allow vDNA synthesis [28–30].

We assume that contrarily to opened/partially opened cores, closed ones will not permit the diffusion of complete ribosomes and of the multiple initiation factors required for translation from the cell cytoplasm to the inside of viral cores (for a review see reference [49]). Based on structural data of the HIV-1 fullerene cone [46, 47], this assumption is in our view reasonable and some of the data provided afterwards in this work support this contention.

### Primary features of the EURT assay

HIV-1 viral particles incorporating EU-repRNA were produced and used to challenge HeLaP4 cells that express both the CD4 receptor and the CXCR4 co-receptor, as described above.

**Fig 2:**
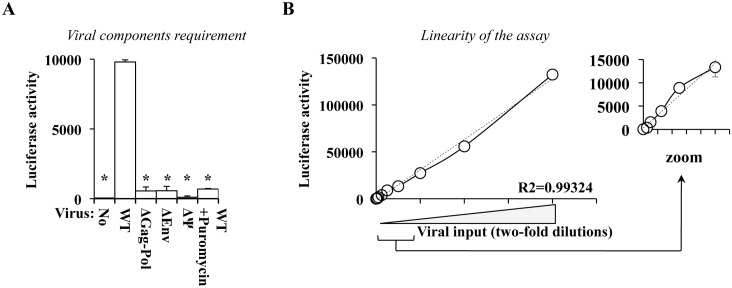
Primary features of the EURT assay. A and B) Reporter HIV-1 virion particles were produced by transient transfection of HEK293T cells with DNAs coding the structural proteins Gag-Pro-Pol and Env (NL4-3), along with EU-repRNA. Supernatants were then ultracentrifuged through a 25% (w/v) sucrose cushion and then used to challenge HeLaP4 cells that bear the appropriate HIV-1 receptor and co-receptor (CD4 and CXCR4). Infection was synchronized and F-Luc activity measured 8 hours post challenge, as depicted in Fig 1A) As control, transfections in which one viral element was omitted were also similarly prepared and used on target cells (Δ, as indicated). To exclude the presence of protein carry-over contaminating the viral preparations, cells were also challenged with WT virus in the presence of the translation inhibitor Puromycin (10 μg/mL). B) To assess the linearity of this assay, cells were challenged with two-fold dilutions of WT HIV-1 reporter virus (provided at MOI-equivalents comprised between 0.0025 and 1, as determined after exo-RT comparison against standards of known infectious titer). Dotted line and R2 value: linear regression analysis of the results. The small graph on the right expands data obtained with lower viral inputs. All graphs present averages and SEM obtained with 4 to 6 independent experiments. * p≤0.05 after a Student t test between WT and the indicated conditions.

To determine whether luciferase accumulation after cell challenge was due to *bona fide* viral particles entry, parallel transfections of HEK293T cells were carried out omitting each one of the three viral components: the Envelope protein (ΔEnv), the Gag-Pol (ΔGag-Pol), or the packaging sequence on the reporter mRNA (ΔΨ). The supernatants were then purified by ultracentrifugation as described above and used at equal volumes on target cells (Fig 2A). Under these conditions, a strong luciferase accumulation was measurable only following challenge with *bona fide* viral particles, but not with preparations devoid of either Env, Ψ or Gag-Pol. Importantly, this assay yielded extremely low background signals (of the order of 5% of the value obtained with WT virus). To further exclude the possibility of luciferase protein carry-over from the initial phase of virion production and purification, a phenomenon known as pseudo-transduction, WT reporter HIV-1 particles were used to challenge HeLaP4 cells in the presence of Puromycin, drug that inhibits *de novo* protein translation. Under these conditions, only background levels of luciferase accumulated in target cells, indicating that *de novo* translation from EU-repRNA delivered by viral particles was absolutely required.

To determine the linearity of the assay, cells were challenged with serial dilutions (two-fold) of WT viral particles prior to cell lysis and F-Luc quantification (Fig 2B, from multiplicity of infection, MOI-equivalents comprised between 0.0025 to 1, as estimated by exogenous-RT activity against standards of known infectivity). The results obtained here indicated that the assay is linear, at least within the range of viral inputs used here.

### The EURT assay yields results that are equivalent to those obtained with the gold-standard assay in HIV entry, Blam-Vpr

The most widely used assay to measure HIV entry relies on the incorporation of a Blam-Vpr fusion protein in HIV particles that, once liberated in target cells, is able to cleave a fluorescence resonance energy transfer (FRET) substrate, CCF2 [36]. Here, we compared the two assays with respect to: receptor-mediated discrimination of viral entry and dose-dependent measurement of viral entry inhibition.

**Fig 3:**
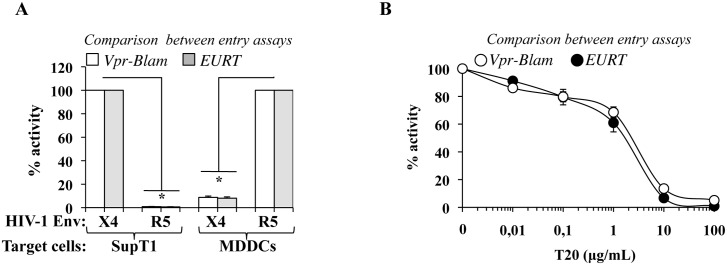
Comparison of the EURT assay with the gold standard in HIV entry, the Blam-Vpr assay. To compare the EURT assay with the widely used Blam-Vpr assay, virions were produced by transfection of HEK293T cells using either reporters. A) Viruses were produced with an R5- or an X4- tropic Env (JR-FL and NL4-3, respectively), then used to challenge SupT1 and monocyte-derived dendritic cells (MDDCs, derived upon incubation of blood monocytes with GM-CSF and IL4 for 4 days). Envelope-receptor specificity was then measured according to the two viral entry assays in lymphocytes (SupT1) and in myeloid cells (MDDCs) either after cell lysis and luciferase activity measurement, or following the incubation of target cells with the fluorescent dye CCF2 and flow cytometry. B) SupT1 were used as target cells to compare the inhibitory rates that could be retrieved upon usage of Vpr-Blam- or EU-repRNA bearing HIV-1 X4 particles in the presence of the HIV-1 fusion inhibitor T20. The graphs present averages and SEM obtained with 3 independent experiments. * p≤0.05 after a Student t test between WT and the respective indicated conditions.

HIV-1 viral particles incorporating either Blam-Vpr or EU-repRNA were produced by transient transfection of HEK293T cells in the presence of an X4- or an R5-tropic envelope protein (NL4-3 and JR-FL, respectively). Virion particles were purified by ultracentrifugation then normalized by exo-RT against standards of known infectivity prior to challenge of SupT1 cells (T cell line) or primary monocyte-derived dendritic cells (MDDCs) (Fig 3A). Under these conditions, virion particles bearing an R5-tropic envelope were unable to enter into SupT1 and conversely, X4-tropic Env bearing particles were drastically diminished in their ability to enter MDDCs, irrespectively of the assay used. Not unexpectedly, the amount of luciferase accumulated following EURT assay in SupT1 was superior to the one measured in MDDCs (S1 Fig). We believe this is due to either a lower proportion of viral particles accessing the cell cytoplasm of MDDCs over SupT1, to their higher degradative activity, to a cell type specific translational rate, or most likely, to a combination of all the above. However, when compared after normalization in each cell type, receptor-mediated discrimination of viral entry was clear.

Next, the susceptibility to T20, an Env-specific entry inhibitor, was assessed in SupT1 during challenge with an X4-tropic HIV-1 virus (Fig 3B). Under these conditions, a clear concentration-dependent inhibition of HIV-1 entry was observed in the presence of T20 following both Blam-Vpr and EURT assays.

Overall, these results indicate that the EURT assay developed here yields result that are perfectly comparable to the widely used Blam-Vpr assay. Thanks to its luciferase readout, it is likely that this entry assay is more amenable for large screens intended to identify novel HIV entry inhibitors.

### Viral core destabilization increases luciferase activity measured following the EURT assay

The process of uncoating, whereby most of CA is shed from viral cores, has been either hypothesized to occur concomitantly with, or after the completion of, reverse transcription. Given that the EU-repRNA is devoid of elements required to initiate this process, the very accumulation of luciferase activity would indicate that either this genomic RNA mimic is incorporated outside viral cores (in which case the assay will simply measure viral entry after viral to cellular membrane fusion), or that either all or a fraction of viral cores are capable of opening up in the absence of reverse transcription.

**Fig 4:**
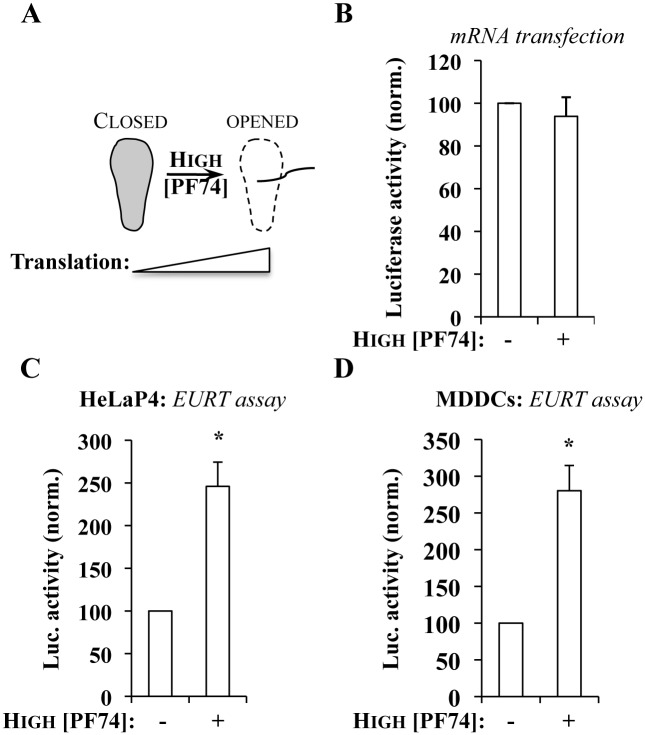
At high concentrations, the HIV-1 core-destabilizing compound PF74 induces an increase in Luciferase activity measured upon EURT assay. A) Due to its well described destabilizing effect on viral cores, PF74 is expected at doses superior to 10 μM to increase the availability of EU-repRNA to the translation machinery. B) To exclude pleiotropic effects of PF74 on translation, HeLaP4 cells were transfected with an *in vitro* synthesized, capped mRNA coding F-Luc in the presence or absence of PF74 (at 10 μg/mL, which corresponds to 23 μM). Luciferase accumulation was measured 8 hours afterwards. C and D) HeLa P4 or MDDCs were challenged with HIV-1 virus, bearing either an X4- or an R5-tropic Env and incorporating EU-repRNA (MOI equivalent of 0.5, after exo-RT normalization against standards of known infectious titer) in the presence or absence of PF74 (as above), prior to cell lysis and Luciferase activity measurement. Averages and SEM of 6 independent experiments are presented (with cells obtained from different donors in the case of MDDCs). *statistically significant differences following a Student t test (p≤0.05).

To obtain further insights into this issue, we first took advantage of PF74 [42]. PF74 is a compound that has been extensively employed to study the very early steps of the viral life cycle, as it binds to a well-defined pocket in capsid hexamers [50]. At high concentrations (≥10 μM), incubation with PF74 inhibits viral infection prior to reverse transcription and leads to the destabilization of viral cores [5, 24, 41, 51, 52]. We surmised that if EU-repRNA is not enclosed in viral cores or if all viral cores behave as a single open entity (i.e. are accessible to the translation machinery), high doses of PF74 will not modify the extent of luciferase accumulation following the EURT assay. On the contrary, if a portion of EU-repRNA were contained within closed cores, their destabilization by PF74 would potentially expose the viral RNA to translation, resulting in an increase in luciferase accumulation (schematically presented in Fig 4A). To determine whether PF74 could modulate translation in a non-capsid dependent manner, cells were transfected with *in vitro* synthesized mRNAs bearing F-Luc in the presence or absence of high doses of PF74 (23 μM = 10 μg/mL), prior to cell lysis and luciferase measurement (Fig 4B). Under these conditions, no notable changes were observed in luciferase activity, indicating that PF74 does not affect translation *per se*. Next, HIV-1 viral particles bearing EU-repRNA and either an X4-tropic or an R5-tropic envelope protein (NL4-3 and JR-FL, respectively), were used to challenge either HeLaP4 cells or primary monocyte-derived dendritic cells (MDDCs) in the continuous presence or absence of PF74, prior to cell lysis and luciferase measurement (Fig 4C and 4D, respectively and S2 Fig for non-normalized values). As expected, PF74 treatment potently inhibited infection with single-round infection competent viruses bearing a GFP reporter that is expressed after integration of proviral DNA (S3 Fig). Using the same conditions, PF74 increased the amount of F-Luc accumulated following the EURT assay by 2.4 to 2.8 fold over control infections carried out in the absence of the compound in both HeLaP4 and MDDCs (Fig 4C and 4D). Given the absence of pleiotropic effects on translation *per se*, these effects must be ascribed to the destabilization of intact viral cores by PF74 and to the concomitant increase in the accessibility of genomic RNA to translation.

Overall, the EURT assay coupled with the use of high doses of PF74, described to destabilize viral cores, indicates that the availability of incoming vRNA to the translational machinery is regulated, at least as it can be measured here, through a PF74-independent mechanism, as well as through a PF74-dependent one. The relative proportions of these activities can be easily estimated as shown in S2 Fig.

### Destabilization of *in vitro* purified HIV-1 viral cores containing EU-repRNA also induces increased luciferase accumulation

**Fig 5:**
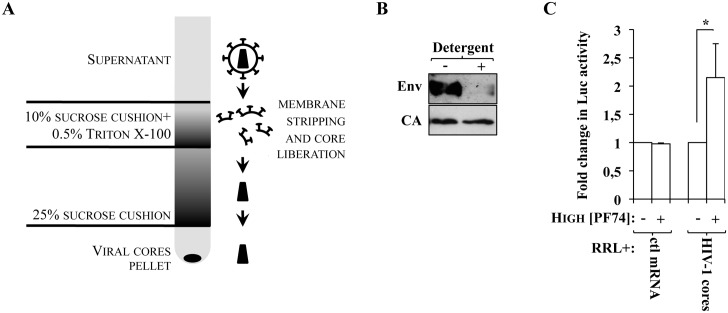
PF74 induces increased Eu-repRNA availability to translation in purified HIV-1 cores *in vitro*. A) Cartoon presenting the experimental setup used to purify viral cores. Briefly, virion particles are passed by ultracentrifugation first through a 10% sucrose cushion containing detergent that deprives the virus of its membrane, and then through a 25% sucrose cushion through which viral cores pellet. Due to their fragility, viral cores are then either immediately analyzed by WB (B) or resuspended in rabbit reticulocyte lysate (RRL) in the presence of PF74 for 30 minutes prior to Luciferase measurement (C). The panel and the graph present data obtained from 3 independent experiments. As control for possible effects of PF74 on RRL activity, a control mRNA coding Luc (ctl mRNA) was used in parallel. *statistically significant differences following a Student t test (p≤0.05).

To further strengthen the observation that the higher accumulation of luciferase observed in the presence of high doses of PF74 was due to viral core opening, we isolated viral cores *in vitro* and determined the behavior of virion-packaged EU-repRNA in an *in vitro* translation system (rabbit reticulocytes lysate, RRL). Viral cores were obtained by ultracentrifugation of viral particles through a layer of 10% sucrose containing 0.5% of Triton X-100 detergent (that removes the viral membrane, as well as membrane-associated proteins, ie Env), placed over a 25% sucrose cushion that then separates solubilized material from viral cores, according to the scheme presented in Fig 5A and as described in [1, 20]. Accordingly, viral core pellets obtained in the presence of detergent are devoid of membrane-associated Env, but contain CA (Fig 5B). Core pellets obtained after this procedure were immediately resuspended in RRL in the presence or absence of PF74 for 30 minutes prior to luciferase measurement (Fig 5C). Under these conditions, PF74 induced a statistically significant increase in the amount of luciferase activity (a 2.15 fold increase over control on average), while no effects were observed on the accumulation of luciferase using an *in vitro* synthesized control mRNA. Overall, these results indicate that viral capsid destabilization leads to an increase in luciferase activity also in a minimal *in vitro* system that uses purified viral cores.

### Luciferase accumulation following the EURT assay is decreased by *cis* mutations in Gag that stabilize viral capsids, while it is increased in the presence of the core-destabilizing restriction factor Trim-CypA

**Fig 6:**
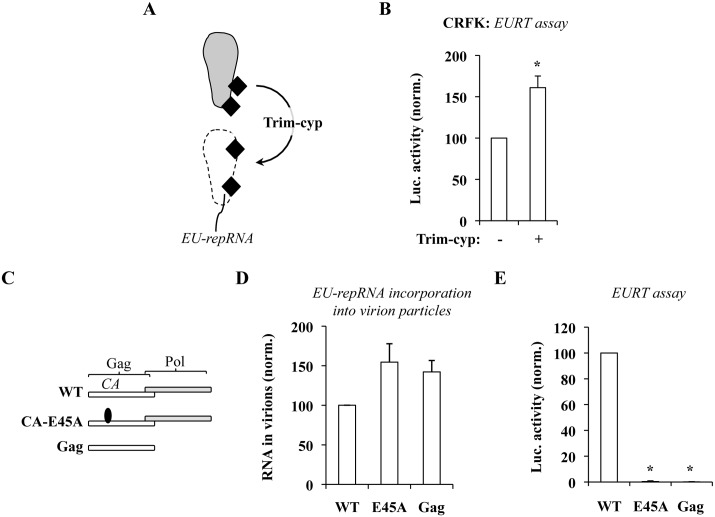
Luciferase accumulation following the EURT assay can be modulated by the status of the viral core. A) Simplified representation of the activity of Owl monkeys Trim-Cyp on HIV-1 capsids after their entry into target cells. B) HIV-1 particles were used to challenge CRFK cells stably expressing or not Trim-Cyp. Given that they lack the appropriate HIV receptors, CRFK cells were priorly transfected with DNAs coding CD4 and CXCR4. C) Scheme of the Gag mutants used here. NL4-3 Env+ and EU-repRNA+ HIV-1 derived viruses were produced by transient transfection of HEK293T cells using the indicated structural proteins. Virions were then purified by ultracentrifugation and normalized by protein content. D) EU-repRNA incorporation into viral particles was determined by lyzing protein normalized virions, followed by nucleic acid transfer onto a nylon membrane, hybridization with a ^32^P-labelled specific probe and phosphor imager quantification. E) The same amount of viral particles used in D was then used to challenge HeLaP4 cells in an EURT assay. The graphs present data obtained with 3 independent experiments. *statistically significant differences following a Student t test (p≤0.05).

An additional approach was then taken to support the argument that the accessibility of vRNA to the translation machinery is influenced by the status of viral cores. Owl Monkeys Trim-CypA (Trim-Cyp) is a restriction factor that potently targets viral cores leading to a consistent impairment of viral infectivity (as schematically presented in Fig 6A) [37, 53–55]. Structural analyses carried out in different laboratories indicate that this restriction factor targets what would fall in our experimental definition of closed cores, i.e. well-ordered fullerene cone structures [56, 57].

Stable CRFK cells expressing Trim-Cyp were obtained upon retroviral-mediated transduction. CRFK are feline cells that lack endogenous Trim expression and are used in the field to study the effects of members of the Trim family on the infectivity of HIV [58, 59]. CD4 and CXCR4 coding DNAs were transiently transfected two days prior to the EURT assay (Fig 6B). Under these conditions, cells expressing Trim-cyp were highly restrictive to parallel infections conducted with single-round infection competent HIV-1 virions that express GFP after viral integration (S4 Fig). However following the EURT assay, luciferase accumulation was more elevated in Trim-Cyp expressing cells than in control ones (1.6 fold, Fig 6B). This result can be explained by the destabilizing action that Trim-Cyp exerts on closed HIV-1 cores and therefore to a concomitant increase in the availability of viral mRNA to translation.

In a complementary approach we sought to negatively modulate luciferase accumulation by increasing viral cores stability. Toward this aim, viral particles incorporating EU-repRNA were produced in the presence of two Gag-Pol mutants: Gag, in which Pol had been removed resulting in the production of immature and highly stable virion-like particles and CA-E45A, in which a single point mutation was introduced in CA (residue E45) in the context of an otherwise *wild type* Gag-Pol polyprotein (Fig 6C). The infectivity of the E45A mutant is severely impaired due to an increased stability of viral cores ([20] and S5A and S5B Fig). Protease-deficient HIV-1 viruses display a decrease in Envelope incorporation of about 8–10 fold and present an entry defect of the same order of magnitude [60], that we also observe for Gag-only virion-like particles following intracellular p24 staining after viral challenge (S5A Fig). However, we reasoned that the high sensitivity of the EURT assay could have potentially allowed us to appreciate defects in addition to the one in viral entry. Virions were produced by transient transfection of HEK293T cells, purified by ultracentrifugation through sucrose and then normalized by exo-RT (or by WB for Gag VLPs). The amount of EU-repRNA incorporated into each viral preparation was determined by slot blot, using a procedure already published [61]. Briefly, equal amounts of viral particles were lysed and immobilized on a nylon membrane with a slot blot apparatus. The amount of RNA was then determined upon hybridization with a ^32^P-labelled specific probe. Under these conditions, no major defects in the amount of virion-associated vRNA were observed and -if anything- mutant virions displayed increased vRNA packaging. When virion particles were used to challenge HeLaP4 cells in the EURT assay, a drastic decrease in the amount of luciferase activity was measured following infection with the CA-E45A single point mutant or with Gag (100 fold, Fig 6E), supporting the notion that hyperstable cores completely preclude access of the translational machinery to the viral reporter mRNA. Of note, no notable changes in Luciferase activity could be measured for the CA-E45A even in the presence of PF74 (S5C Fig). As previously described, Gag-only virion-like particles displayed an 11 fold entry defect (as estimated by intracellular p24 measurement by FACS three hours post viral challenge, S5A Fig). The 100-fold defect estimated by EURT for this mutant is therefore likely the sum of an entry defect and of an higher protection/retention of the viral genome in hyperstable cores.

Overall, these results indicate that the amount of luciferase measured following infection is influenced by the stability of viral cores.

### Time of addition experiments reveal a progressive loss of susceptibility of viral cores to high concentrations of PF74

Overall, the results gathered so far indicate that the reporter genome used in our assay is contained in at least two measurable species of viral capsids: those directly accessible to translation (defined here as completely or partially opened); and those whose opening requires high doses of PF74 (defined here as closed). To understand the relationship between these species, we took advantage of a previous study carried out by our laboratory focused on a thorough characterization of the kinetic behavior of functional viral genomes during the early phases of HIV infection [62]. As a way to reveal effects of the cellular environment on incoming viruses, we had measured the functional stability of viral cores arrested at their pre-reverse transcription state by carrying out infections with GFP-coding HIV-1 viruses in the presence of a reversible RT-inhibitor (Nevirapine) removed at different times post infection prior to FACS three days later. Without going into the details of the results of this study, the ability to measure the proportion of viral cores that retained their functionality at any given time (i.e. their ability to resume infection and yield GFP-positive cells), allowed us to determine that in the absence of reverse transcription infectious viral cores undergo a rapid inactivation with kinetics that vary according to the cell type and cell stimulation used [62].

**Fig 7:**
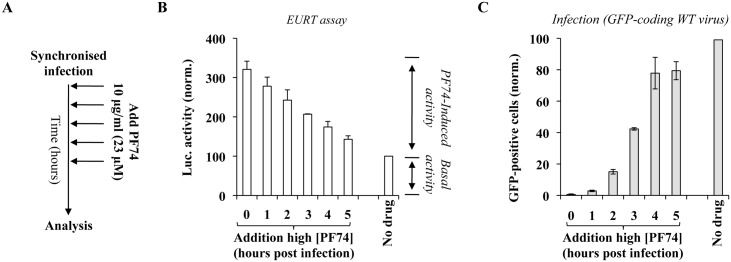
Effects of time dependent addition of high doses of PF74 on EURT and viral infectivity. A) Experimental scheme used here. Depending on the input virus (containing EU-repRNA, or a miniviral genome competent for a single round of infection and coding GFP), cells were either lysed at 10 hours post infection for EURT (B), or analyzed three days afterwards by flow cytometry (C). The graphs present averages and SEM of four independent experiments.

To determine whether the proportion of PF74-dependent luciferase activity changed over time and reflected the behavior of functional viral capsids arrested at their pre-RT state that we had determined previously, viruses were used to challenge HeLaP4 cells in a synchronized infection in the presence or absence of PF74 (23 μM = 10 μg/mL) added at the indicated time post infection prior to luciferase activity measurement (10 hours post infection; Fig 7A and 7B). The amount of luciferase was normalized to the one obtained in the absence of PF74 (Fig 7B). The PF74-induced luciferase activity was maximal when the drug was added at t0 and decreased afterwards, reaching near basal levels (ie levels measured when infections were carried out in the absence of PF74), by 5 hours post infection.

When a similar analysis was carried out with single round of infection competent GFP-coding HIV-1 viruses and the accumulation of GFP-positive cells was measured three days afterwards, a diametrically opposite behavior was observed, so that the complete inhibition observed when PF74 was added at t0 progressively subdued when the compound was added 5 hours after infection (Fig 7C). These results are in complete agreement with several studies in the literature indicating that early addition of high concentrations of PF74 destabilizes viral cores impairing the completion of the early steps of the viral life cycle [5, 51] and implement them by indicating that as a result of this process the viral genome becomes more exposed to the cytoplasmic environment. Viral cores seem to remain susceptible to high doses of PF74 for longer times following EURT, than previous reported in the case of CsA washout experiments in cells expressing Trim-Cyp (≤1 hour, [5]. We believe this may be mostly due to experimental differences, and that a more appropriate comparison could be drawn with our previous study [62] focused on the functional stability of HIV-1 viral complexes arrested at their pre-RT stage (the data presented in Fig 7A are indeed obtained with a reverse transcription incompetent virus). In this case, the time at which half of the PF74-dependent luciferase activity is lost over time (t_50%_, between 3 and 3.5 hours post infection) is in perfect agreement with the one we measured for the functional stability of HIV-1 viral complexes arrested at their pre-RT stage (t_50%_ of 3.8 hours in HeLaP4 cells [62]). Therefore, these data along with data in the literature, strongly suggest that the PF74-induced luciferase activity measured by the EURT assay reflects the behavior of vRNA contained in *functional* viral cores, i.e. cores that contain infectious viral genomes.

### Reverse transcription accelerates the loss of susceptibility of viral capsids to high concentrations of PF74 as measured by EURT

**Fig 8:**
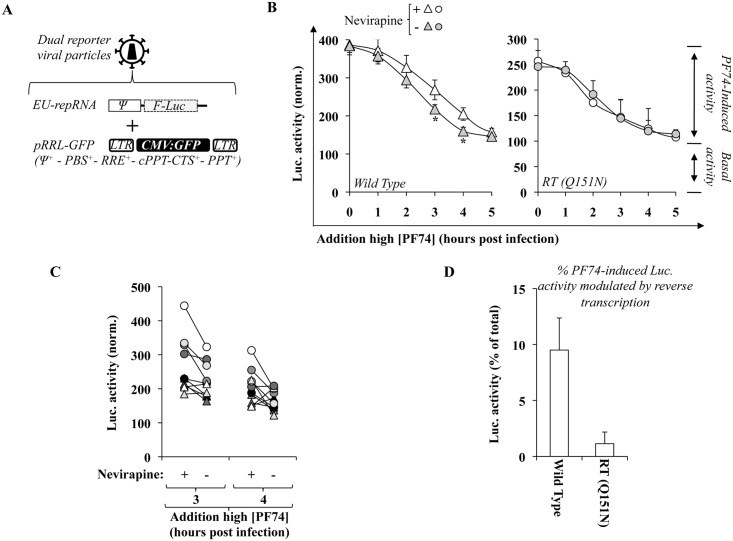
Contribution of reverse transcription to the susceptibility of viral cores to high doses of PF74. A) Dual reporter viral particles were produced by co-transfection of HEK293T cells with DNAs coding Gag-Pol, Env along with the two genomes: EU-repRNA and pRRL-GFP that contains all the elements required for its mobilization in target cells. To favor co-packaging of EU-repRNA in reverse transcription competent viral particles, a ratio of 9 to 1 was used between pRRL and EU-repRNA. *CMV*, cytomegalovirus promoter; *LTR*, long terminal repeats; *Ψ*, packaging sequence; *PBS*, primer binding sequence; *RRE*, Rev-responsive element; *cPPT-CTS*, central polypurine tract-central termination sequence; *PPT*, polypurine tract. B) Dual reporter viral particles containing a wild type or a mutated RT (Q151N) were used to challenge HeLaP4 cells in the presence or absence of Nevirapine (10 μM). PF74 was added at 23 μM at the indicated time post infection and the overall amount of luciferase activity was measured at ten hours post infection. Values are normalized for the ones observed in the absence of PF74. The graphs present AVG and SEM obtained from 4 to 9 independent experiments. *, statistically significant differences following a Student t test (p≤0.05). C) The graph presents the decrease in the amount of PF74-induced luciferase activity in the individual experiments that compose Fig 8B at t3 and t4. D) The graph presents the percentage of PF74-induced luciferase activity lost in reverse transcription competent viruses over reverse transcription incompetent ones (data of Fig 8B, pooling t3 and t4 data points).

Reverse transcription does influence the behavior of viral cores, as it has been measured through different experimental setups, however the nature of these changes is still a matter of debate. Given that the amount of luciferase activity that accumulates following EURT is influenced by the access of viral capsids to the translational machinery, we surmised that additional insights on this issue could be gathered using the assay developed here. Given that the RNase activity inherent to reverse transcription would degrade the reporter RNA if this were directly reverse transcribed, we segregated reporter and reverse transcription functions on two separate genomes co-packaged in the same viral particle. To this end, viral particles were produced by transfection of HEK293T cells in the presence of EU-repRNA and of a miniviral genome bearing *gfp* and competent for both reverse transcription and integration (pRRL-GFP) provided in large excess to favor encapsidation of EU-repRNA in reverse transcription-competent virion particles (Fig 8A, 9:1 ratio, after optimization experiments). Co-expression of two viral genomes in producing cells is not proof of their co-packaging in the same viral particles, so that the possibility exists that EU-repRNA and pRRL-GFP segregate on distinct particles. However as shown afterwards, reverse transcription drives changes that are measurable by EURT, supporting the notion that at least a measurable fraction of viral genomes are co-packaged within the same viral particle.

Dual reporter viruses were produced as described above and then used to challenge recipient cells in the presence or absence of the RT inhibitor Nevirapine. PF74 was then added at different times to determine the kinetics of the loss of susceptibility of PF74-induced luciferase activity in RT-competent and -incompetent viruses following infection. Under these conditions, the amount of PF74-induced luciferase activity measured following WT virus infection decreased more rapidly in the absence of Nevirapine than in its presence at 3 and 4 hours post infection, indicating that reverse transcription contributed to the more rapid loss of susceptibility of viral capsids to high concentrations of PF74 (Fig 8B, t_50%_values of 2.5 hours). To determine whether this loss was truly dependent on reverse transcription, the same experiment was carried out using a single point mutant in RT, severely hampered in its ability to support reverse transcription (Q151N, Fig 8B, right panel [62]). For reasons that are unclear at the moment, the relative proportion of PF74-induced activity over basal one was lower than for the WT. However, no changes were observed in the kinetics in the presence or absence of Nevirapine, as expected given that this mutant is already reverse transcription impaired.

Therefore, reverse transcription is responsible for the faster loss of susceptibility of viral cores to high concentrations of PF74 as measured by EURT. The changes measured here within the five hours window of infection are subtle yet clearly significant at 3 and 4 hours post infection (Fig 8C details the decrease in the proportion of PF74-induced activity across individual experiments).

When taking into account the fact that EURT yields a measure of the entire spectrum of viral cores present during infection (functional and non-functional), we believe it possible to estimate the fraction of PF74-induced luciferase activity modulated by reverse transcription to reveal the proportion of viral capsids that undergo structural changes (by subtraction of the values measured in the absence of Nevirapine, to those obtained in its absence at the same time point). Our results indicate that 9.5% of the PF74-induced luciferase activity is lost on average following *wild type* HIV-1 infection (Fig 8D). This estimate approximates rather closely the proportion of infectious viral genomes that has been measured using different experimental setups (1 infectious for 8 non-infectious ones, [5, 51]).

### Effects of different concentrations of PF74 on EURT

**Fig 9:**
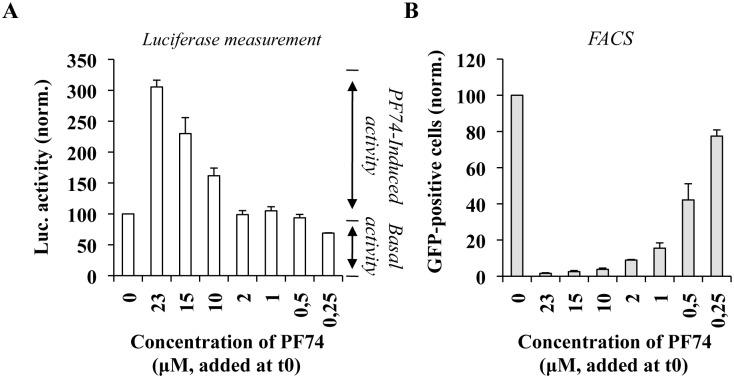
Determination of the effects of different concentrations of PF74 on EURT and viral infectivity. Dual reporter viruses were used to challenge HeLaP4 cells and either left untreated or treated at t0 with the indicated concentration of PF74. The extent of luciferase activity was measured eight hours post infection (A), while the percentage of GFP-positive cells was determined three days later by flow cytometry (B). The graph presents data obtained from 5 independent experiments.

PF74 is a potent inhibitor of the early phases of the viral life cycle, but it has been shown to exert different effects during infection, most notably prior or after the completion of reverse transcription depending on its concentration. At the high concentrations used here up to this moment (≥ 10 μM), PF74 destructures viral cores and inhibits the early phases of infection prior to reverse transcription. On the contrary, at lower concentrations of PF74 (2 μM) reverse transcription is completed, although infection is still inhibited [5, 51]. We reasoned therefore that the EURT assay could be used to determine whether different concentrations of PF74 result in differential exposure of the viral genome, potentially linking uncoating and the changes induced by reverse transcription as they can be measured classically through the loss of viral components, to the exposure of the viral genome contained in viral cores to the cellular environment. To this end, infections were carried out with dual reporter particles, as before in the presence of different amounts of PF74 provided at t0 of infection (Fig 9A). As shown before, high concentrations of PF74 (23, 15 and 10 μM) induced a measurable increase in the amount of luciferase when compared to control infections carried out in the absence of compound. However, no changes in luciferase activity were measured in the presence of concentrations of PF74 equal or lower to 2 μM. In agreement with previous data, PF74 exerted a strong inhibitory effect on the completion of the early phases of the viral life cycle at concentrations ≥ 2 μM, as measured by flow cytometry-mediated determination of the percentage of GFP-positive cells three days post infection (Fig 9B).

Although we have not directly measured the extent of reverse transcription here, the concentration of 2 μM of PF74 have been clearly established to impair the viral life cycle after reverse transcription [5, 51]. Therefore, these results may suggest that despite the fact that reverse transcription induces structural changes that can be measured (for instance in this study, via changes in the susceptibility to high doses of PF74, as in Fig 8B), restructured viral cores may still retain their ability to protect the viral genome from the cellular environment.

### The kinetics of accumulation of luciferase activity following EURT support the contention that reverse transcription does not increase the exposure of the viral genome to the cytoplasmic environment

**Fig 10:**
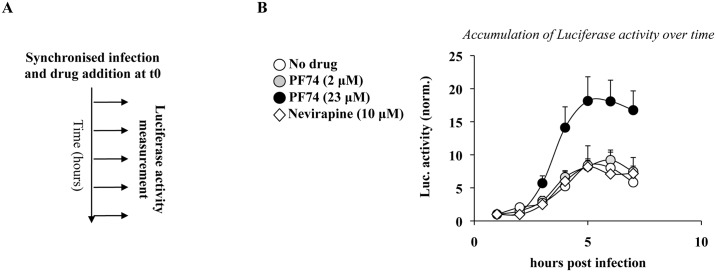
Time dependent accumulation of Luciferase over time and influence of Nevirapine and different concentrations of PF74. A) To determine the extent to which reverse transcription could expose the viral genome and therefore increase the accumulation of Luciferase activity, dual reporter and reverse transcription competent viral particles were used to challenge HeLaP4 cells according to the scheme provided in A. B) For each condition, the amount of Luciferase that accumulated over time is provided after normalization to the activity measured 1 hour post infection. The graph presents AVG and SEM of 8 independent experiments.

To further determine whether reverse transcription could increase the exposure of the viral genome to the cytoplasm, the accumulation of luciferase activity over time was examined during infections with the dual reporter viruses described in Fig 8. Infections were carried out with WT virus incubated at t0 (according to the scheme of Fig 10A) with: no drug and Nevirapine (reverse transcription competent or not, respectively); PF74 at 23 and 2 μM (that inhibit infection prior or after reverse transcription). Under these conditions, luciferase activity accumulated over time for all conditions and consistent with its core-opening effects, higher luciferase activity was measured in infections carried out in the presence of high doses of PF74. Surprisingly however, the luciferase activity measured in the presence of 2 μM PF74 was identical to the one measured in the presence or absence of Nevirapine.

Therefore, reverse transcription, or conditions that allow its completion are clearly not sufficient to expose the viral genome to the cellular environment. In our view, these results are important as they fill the gap existing between the mechanical loss of viral components from viral cores (CA for instance) and the consequences that viral core restructuration may have for the interaction of the viral genome with its surrounding environment.

### The EURT assay reveals a capsid destabilizing activity in MDDCs treated with IFNα

IFNα exerts a negative effect on HIV-1 infection and particularly on reverse transcription [63–65]. However, since this process is highly receptive to conditions that modify the properties of viral capsids [20], we sought to determine whether IFNα treatment could modify the proportions of PF74-dependent/independent luciferase activity measured by the EURT assay, as manner to apprehend possible effects of IFNα on viral cores. To this end, HeLaP4 and MDDCs in which HIV-1 infection is respectively resistant or susceptible to IFN were incubated for 24 hours with the indicated amount of IFNα prior to viral challenge. As expected, HIV-1 infection of HeLaP4 was resistant to IFNα, while the one of MDDCs displayed a dose dependent susceptibility to IFN when using a GFP coding virus (S6 Fig). Next, identical conditions were used for EURT.

**Fig 11:**
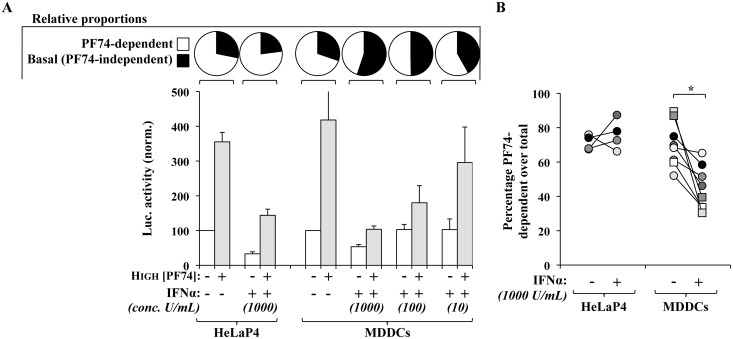
IFNα-treatment of MDDCs decreases the proportion of PF74-dependent luciferase activity that can be measured following EURT assay. A) HIV-1 viral particles bearing X4- or R5-tropic envelopes were used to challenge either HeLa P4 or primary MDDCs treated for 24 hours prior to viral challenge with the indicated concentration of IFNα. Infections were carried out in the presence or absence of PF74 (10 μg/mL, 23 μM). Luciferase accumulation was then measured as described before. Averages and SEM are presented here after normalization to the values obtained in untreated cells. The pie charts above the graph present the relative proportions of PF74-dependent and independent activities measured in the presence or absence of IFNα. B) The graph present individual donors (n = 8 for MDDCs and 4 independent experiments for HeLaP4 cells) that were used in the left panel, with a focus on the decrease in the percentage of PF74-dependent activity measured following incubation with IFNα. *statistically significant differences following a Student t test (p≤0.05).

IFNα is known to exert a negative effect on translation (recently reviewed in [66]) and indeed, the basal levels of luciferase measured following EURT assay in cells treated with IFNα decreased with respect to untreated controls (Fig 11A). However, after normalization for this effect in each condition (in IFNα-treated or -untreated cells), IFNα inverted the proportions between PF74-induced and basal luciferase activity and the fraction of the former dropped from 75% of the total to 34% (Fig 11A, pie charts). The extent of loss of PF74-dependent activity was dependent from the dose of IFNα used and inversely correlated with its overall antiviral effect.

Not surprisingly, the magnitude of this effect varied among donors although IFNα stimulation of MDDCs, but not HeLaP4 cells, invariably led to a decrease in the proportion of PF74-dependent luciferase activity to the benefit of the PF74-independent one (Fig 11B). Although this effect is not as drastic as the infectivity defect that can be measured by flow cytometry, it is nonetheless reproducibly observed in cells of different donors and it must be remembered that infectivity and EURT assays rely on different experimental settings and timeframes (3 days *versus* 8 hours).

Overall, the results presented here suggest that IFN-stimulation is able to decrease the proportion of PF74-dependent luciferase activity that accumulates following EURT assay to the benefit of the PF74-independent one. We hypothesize that these results underlay the existence of a cellular activity that destabilizes, or directly degrades, viral cores in IFN-treated MDDCs and that may be at the origins of the infectivity defect measured in these cells.

## Discussion

In the work presented here, we aimed in the first instance at developing a novel HIV-1 entry assay based on a viral genomic RNA mimic delivered and directly translated into the cell cytoplasm after viral to cellular membrane fusion. The data we present here indicate that in its easiest setting, this assay is highly sensitive, it is linear and yields virtually no background when HIV-1 viral particles are produced and purified as described here. Side-by-side comparison with the more widely used Blam-Vpr based test [36] points to the EURT assay as a valid alternative to study viral entry, at least for most applications (see further considerations below). We believe that in light of the fact that it uses a luciferase-based readout, the assay developed here may be easier to handle large screens that aim at identifying novel inhibitors of HIV-1 entry.

However, while the Blam-Vpr assay relies on the direct liberation of a virion-incorporated enzyme that is loosely associated to viral cores [36, 67], the EURT test relies on the translation of a core-packaged RNA and this property is influenced not solely by viral-to-cellular membrane fusion, but also by cell type specific translation or RNA degradation rates, as well as by the very conformation of viral capsids that contain the reporter RNA. We believe that these caveats, if carefully considered, can be either corrected for (introducing appropriate normalization controls, as we have done here to compare SupT1 and MDDCs), or can be exploited to yield additional information on the behavior of viral capsids from a point of view that is rather understudied so far: the degree of exposure of the viral genome to the cell cytoplasm. If susceptible to the relatively *bulky* process of translation (multiple ribosomes, initiation and termination factors), the viral genome contained in these cores must be visible to a plethora of cellular effectors, among which antiviral nucleases and sensors.

The EURT assay relies on the accumulation of luciferase activity and it shall be conceded that this is an indirect measure of the status of viral cores. Yet, several lines of evidence support the notion that the rates of luciferase translation from the reporter RNA strictly depend on the behavior of viral cores. First, infections carried out in the presence of high concentrations of PF74, a well characterized core-destabilizing agent, lead to increased accumulation of luciferase. Second, this effect can be reproduced *in vitro* using isolated viral cores. Third, similar effects are observed with Trim-Cyp that also associates to and destabilizes capsid lattices [37, 53–55]. Fourth, at the opposite of this behavior hyperstable viral cores completely impair the accumulation of luciferase. While Gag-only VLPs constitute a rather artificial setting, the single point mutation E45A in CA leads to *bona fide* hyperstable capsids that have been well characterized biochemically [20, 30].

By comparing infections carried out in the absence or presence of high doses of PF74 that artificially disrupt HIV capsids, the EURT assay allowed us to appreciate for the first time the existence of two quantifiable species of viral cores: those in which the core-packaged reporter RNA is readily accessible for translation (PF74-independent or basal) and those in which access to RNA requires artificial capsid destabilization (PF74-dependent). The opposite behavior observed in time of addition experiments between the positive effects that PF74 exerts on luciferase activity and the negative ones exerted on the accumulation of GFP-positive cells, in addition to the similar rates of decay of functional viral capsids frozen at their pre-RT state that we had previously measured [62], strongly suggest that the PF74-dependent luciferase activity results from RNA enclosed into *functional* viral capsids, while the basal activity results from *non-functional* opened cores presenting unrestricted access to cytoplasmic effectors.

In this study, we define opened and closed cores, uniquely in reference to their accessibility to the translational machinery. Of course this experimental distinction focuses on the main measurable capsid species and will likely miss intermediate/transient ones. Given the structural and biochemical data available, it is not hard to envision that the ordered fullerene cone structure, that would fall into our definition of closed core, represents an obstacle to the free diffusion of ribosomes, as well as of translation initiation and termination complexes. However, viral cores that have lost most of their capsid, as uncoated cores are mostly described to do, may allow or still prevent access of the translational machinery (referred to as open or closed in this study).

This specific aspect is largely, if not entirely, unappreciated. In particular, it is unknown whether uncoating (and all the structural and biochemical changes associated to this process) result in the formation of novel viral nucleoprotein structures that still retain their ability to protect the viral genome from an hostile environment, or whether on the contrary they allow unrestricted access to cytoplasmic proteins. From the point of view of how viral infection is perceived by the cell and of how the virus may prevent cellular detection, this aspect may be a key determining factor in viral infection.

Reverse transcription induces changes that we can also measure here as a faster loss of susceptibility of viral cores to high doses of PF74. These changes may not appear major when compared to other assays [5, 29, 51]. Yet, they are statistically significant in an experimental setup that first of all considers all the viral species together (both functional and non-functional, therefore diluting overall differences) and that is limited to the very first hours of infection. Therefore, on the whole, this data is clearly in line with experimental evidence gathered with other assays indicating that reverse transcription modifies the behavior of viral cores. However, we show here that reverse transcription does not result in an increased exposure of the viral genome to the cell cytoplasm (i.e. viral cores remain in what we define here as closed form). The simplest and most straightforward experiment in support of this contention is that luciferase accumulates similarly over time in the presence or absence of nevirapine, as well as in the presence of low doses of PF74 (2 μM) that have been clearly described in the literature to inhibit viral infection after reverse transcription [5, 51]. Therefore, our data support the notion well supported in the literature that reverse transcription reshapes viral cores [3, 5, 7, 30, 68], but also indicates that even so, viral nucleoprotein complexes still retain their ability to withdraw the viral genome from an hostile cellular environment.

Interestingly, the results obtained in this study indicate that an average of 34% of viral particles entering target cells (this proportion ranges from 20 to 52% across 20 different viral preparations with an average of 34%) are composed of cores that are intrinsically opened and exposed to the cytoplasmic environment. In non-stimulated cells, the proportion between the two species remains constant despite differences in the susceptibility of target cells to infection (HeLa cells and MDDCs are respectively susceptible and resistant to HIV infection ([69]. Therefore, we believe these results are more likely to reflect an initial heterogeneity in the viral population [1, 35], perhaps due to improper assembly. However, this heterogeneity may serve a purpose during the subsequent cycle of infection, for example as a decoy for cellular restriction factors. We do not believe these non-functional cores will play a part in the sensing of HIV-1 infection, given the likelihood that they will present viral mRNA, not DNA to the cell and that retroviral genomic RNA mimics cellular mRNAs.

However, upon IFNα-stimulation of IFN-sensitive cells (MDDCs), the proportion of closed cores shifts towards opened ones in a clearly measurable manner. IFNα exerts numerous effects on the cell physiology and these differences could in principle be due to an effect at viral entry via the modulation of cellular receptors, or to an effect on translation *per se*. However, the drift from closed to open viral cores is apparent after correction for these factors, indicating that it likely reflects true behavioral changes of viral cores in an IFN-environment.

IFNα treatment of MDDCs inhibits viral infection mostly at reverse transcription, but this process is heavily influenced by the status of viral capsids. We believe therefore likely that the shift observed between the different viral core populations underscores an IFN-effector driven phase of destabilization and/or degradation that culminates into the larger defects observed in reverse transcription under these conditions.

In conclusion, the assay developed here allows for the first time to appreciate the complex dynamics of HIV-1 viral nucleoprotein complexes from the point of view of viral genomic RNA with a particular focus on its degree of exposure to the cytoplasmic environment. We believe this assay can be used to shed further light on the very early steps of the intracellular life of HIV-1.

## Materials and Methods

### Plasmids

Plasmids required for the production of HIV-1-based lentiviral vectors capable of a single cycle of infection have been described before [70]: 8.2, expressing the structural proteins Gag-Pro-Pol (in addition to Tat and Rev); NL4-3 and JR-FL Envelopes, expressing respectively an X4- and an R5-tropic HIV-1 Envelope; the HIV-1 Rev protein, pRRL-GFP, a miniviral HIV-1 genome comprehensive of all the *cis*-elements required for its reverse transcription and integration (LTRs, PBS, RRE, cPPT-CTS and PPT) and bearing a CMV:GFP expression cassette that allows the identification of infected cells by flow cytometry.

The Eu-repRNA reporter plasmid was engineered by appending a minimal packaging sequence of HIV-1 (Ψ, nt 205–338, HIV-1 strain BRUCG, #K02013, according to [71], upstream of the Firefly Luciferase (F-Luc) open reading frame in pcDNA3.1+ (Invitrogen) by standard molecular biology techniques. The ΔΨ is an F-Luc expressing construct devoid of Ψ.

A Rev-independent HIV-1 Gag expression construct that allows the formation of immature viral particles composed of unprocessed p55 Gag in the absence of other viral proteins has been described in [72].

CA-E45A contains a single point mutation in the HIV-1 CA in the context of an otherwise wild type Gag-Pro-Pol [20]. This single point mutation in CA drives the formation of hyperstable cores and was obtained by Felipe Diaz Griffero (Albert Einstein College of Medicine, Bronx, NY, USA). The QI51N RT mutant has been described before [62].

EXN-TrimCyp expressing the Owl monkey Trim-cyp fusion protein has been obtained from Greg Towers (University College of London, London, UK) and has been described in reference [73]. This MLV-based construct allows for the retroviral-mediated expression of the protein of interest. Upon transduction, expressing cells can be selected thanks to the Neomycin selection carried by the vector.

CD4 and CXCR4 coding plasmids were obtained through the AIDS Reagent and Reference Program of the NIH.

The Blam-Vpr coding plasmid has been described before [36].

### Cells, antibodies, cytokines and chemical compounds

HEK293T, HeLaP4 cells (expressing CD4 along with CXCR4) were maintained in complete DMEM media supplemented with 10%FCS (both cell lines were obtained from CelluloNet, SFR Biosciences-Gerland). The T cell line SupT1 (CelluloNet, SFR Biosciences-Gerland) was instead propagated in RPMI1640 media and 10%FCS. Immature monocyte-derived dendritic cells (MDDCs) were obtained from the blood of healthy donors through the EFS of Lyon [70]. Discarded “leukopacks” (cells discarded from platelet donors) were obtained anonymously so that gender, race, and age of donors are unknown to the investigator and inclusion of women, minorities or children cannot be determined. This research is exempt from approval, although written informed consent was obtained from blood donors to allow use of their cells for research purposes. White blood leukocytes were first purified through successive Ficoll and Percoll gradients. Monocytes were retrieved at the Percoll interface and were further purified by negative depletion (monocyte isolation kit II, catalogue n° 130-091-153, Miltenyi) to obtain a cell population of purity equal/superior to 95%. Monocytes were then differentiated upon incubation for 4 days with GM-CSF and IL4 (provided at a final concentration of 100 ng/mL each, catalogues n° PCYT-221 and PCYT-211, Eurobio). When indicated, cells were incubated for 24 hours with human IFNα prior to viral challenge (catalogue n° 11100–1, Tebu Bio).

PF74 (catalogue n° SML0835-5) and Neomycin were purchased from SIGMA. The fusion inhibitor T20 was obtained from the AIDS reagents and reference program of the NIH.

The anti-CA antibody (clone n° 183-H12-5C) was obtained from the AIDS Reagents and Reference Program of the NIH), while the anti-Env antibody was purchased from AbCam (catalogue n° ab21179).

### Virion production, purification and titration

HIV-1 virion particles were typically produced upon ectopic DNA expression of HEK293T cells with plasmids coding respectively for: the packaging proteins Gag-Pro-Pol, the HIV-1 Rev protein, the desired HIV-1 envelope protein and the packaged viral genome (either the EU-repRNA, for the production of viral particles dedicated to the EURT assay; pRRL-GFP for the production of viral particles competent for a single round of infection; or both, for dual reporter viruses).

In all cases, HEK293T cells were transfected at a 8(Gag-Pro-Pol):8(genome):2(Env):0.5(Rev) ratio for a total of 20 μg per 10cm plate. When indicated, viral particles packaging both EU-repRNA and pRRL-GFP were produced by simultaneous transfection of the two genomes along with the remaining viral components. To maximize the chances of packaging of the EU-repRNA in reverse transcription competent viral particles, a large excess of pRRL-GFP over the reporter EU-repRNA was used (9:1). Optimization experiments had indicated this ratio, as the lowest with which luciferase activity could still be satisfactorily measured following EURT. When indicated, 4 μg of Blam-Vpr coding DNA were added to the ones mentioned above.

Virions were subsequently purified by ultracentrifugation at 25.000 rpm for 2 hours through a 25% sucrose cushion (w/v), resuspended and titered. The number of infectious viral particles was directly measured upon challenge of HeLaP4 cells with dilutions of the viral preparations, followed by flow cytometry analysis three days after infection (in the case of GFP coding viruses). All other viruses were quantified by exogenous-RT activity (Exo-RT) and the number of infectious viral particles was estimated by comparison with viruses of known infectivity. Exo-RT measures the ability of particles-associated reverse transcriptase enzyme (RT) to incorporate radioactive dTTP in an exogenous RNA:DNA substrate constituted by a poly-rA matrix and by an oligo dT primer. Gag-only virion particles lack RT and were therefore normalized to standard virions by WB. In some instances, an in-house p24 ELISA was also used to normalize viral particles.

### Infections

In the case of EURT assays, HIV-1 viral particles were added to target cells for 2 hours at +4°C, prior to cell washing at +4°C. The temperature was then shifted to 37°C to induce viral entry into the cell. Eight hours post infection (that preliminary experiments identified as the earliest optimal time required for the accumulation of robust luciferase activity), cells were lyzed and F-Luc activity measured according to standard procedures (Promega, catalogue n° E4530, using a Turner Biosystems luminometer). For Blam-Vpr assays, the procedure described in [36] was essentially followed. Briefly, cells were incubated with Blam-Vpr containing virions for 1 to 3 hours at 37°C. Cells were then washed once with CO2-independent medium (Gibco-BRL, Rockville, Md.), and loaded with CCF2/AM dye, as recommended by the manufacturer (Life technologies). Cells were then washed twice with CO2-independent medium and incubated overnight in CO2-independent medium supplemented with 10% FBS and 2.5 mM probenecid to allow CCF2 processing by the Blam-Vpr. The change in emission fluorescence of CCF2 after cleavage was monitored by flow cytometry using a BD FACSCanto II. For intracellular Gag staining, cells were extensively washed after viral challenge, trypsinized to remove external viral particles and then intracellularly stained with an anti p24 antibody conjugated to phycoerythrin that recognizes all major Gag species (Beckman Coulter, #6604667). Data were collected with CellQuest and analyzed with FlowJo software (Treestar, San Carlos, Calif.).

In the case of single cycle infection competent GFP-coding viruses, the extent of infection was instead measured 3 days after infection by flow cytometry. When indicated, PF74 was added at a concentration of 10 μg/mL.

### Viral core purification and translation in RRL

HIV-1 virion particles incorporating EU-repRNA produced by transfection of HEK293T cells as described above were layered on a two step sucrose gradient consisting of a layer of 10% (w/v) sucrose containing or lacking 0.5% Triton X-100 (1 mL) and of one of 25% sucrose (2 mL). Gradients were ultracentrifuged at 28.000rpm for 1h15min at 4°C and after discarding the supernatant, core pellets were either immediately analyzed by Western blot or resuspended in a final volume of 50 μl with nuclease-treated Flex Rabbit Reticulocyte Lysate (Promega) for *in vitro* translation for 30 min at 30°C. When indicated, PF74 (23 μM) was added on core pellets. Control mRNA coding luciferase was included in the RRL kit.

### Quantification of packaged gRNA in virion particles

Virion particles produced, purified and normalized as mentioned above were lysed and then transferred on a nylon membrane using a slot blot apparatus (Bio-Rad). Nucleic acid was then fixed by UV cross-linking and the membrane was hybridized overnight at 42°C with a ^32^P labeled DNA probe specific for EU-repRNA (antisense orientation, nt 319–338 of Ψ, CATCTCTCTCCTTCTAGCCT) in 10% Denhart’s, 1.5x SSPE (1x SSPE is 0.18 M NaCl, 10 mM NaH_2_PO4, and 1 mM EDTA at pH 7.7), 7% SDS and 100 μg/mL of tRNA. The membrane was the thoroughly washed in 0.1% SDS, 0.2x SSC and exposed for phosphorimager analysis. To ensure that the values obtained were within the linear range of the assay, a standard curve was obtained by serial dilution of WT virus, as previously described [61].

## Supporting Information

S1 Fig Non-normalized values of luciferase accumulation following EURT assay in SupT1 versus MDDCs. The data in Fig 3A is presented after normalization to controls to allow the direct comparison between different readouts. The graph above presents instead the straight luciferase values obtained following EURT assay, using the same amount of virion particles on the above-mentioned cell types.
(TIF)

S2 Fig Non-normalized values of luciferase accumulation following EURT assay in HeLaP4 versus MDDCs, example of method used to measure the relative proportions of PF74-dependent and independent activities following EURT and corresponding viral cores behavior. (Left) Non-normalized data related to the graphs of Fig 4C and 4D. (Right) The luciferase activity measured in the presence of PF74 (at 23 μM = 10 μg/mL) includes also the one that can be obtained in the absence of this compound. As such, the relative proportions of PF74-dependent and -independent luciferase accumulation can be measured by subtraction of the basal activity to the overall activity measured during EURT in the presence of PF74. The expected behavior of viral cores with respect to their accession to the translation machinery is also schematically presented.
(TIF)

S3 Fig Effects of PF74 upon HIV-1 infection of HeLa P4 and MDDCs. Monocyte-derived dendritic cells were challenged with a GFP-coding JR-FL Env-bearing HIV-1 vector in the presence or absence of PF74 (at 23 μM = 10μg/mL), prior to flow cytometry analysis 3 days after infection. HeLaP4 were instead challenged with NL4-3 envelope-pseudotyped viral particles. The graph presents data obtained from 6 independent experiments. *statistically significant difference following a Student t test (p≤0.05).
(TIF)

S4 Fig Susceptibility of CRFK cells expressing Owl Monkey Trim-Cyp to HIV-1 infection. Feline CRFK cells were stably transduced with an MLV-based retroviral vector expressing or not the Owl Monkey Trim5-Cyp fusion protein. Upon puromycin selection, cells were transiently transfected with DNAs coding the HIV-1 receptor/co-receptor CD4 and CXCR4, prior to single round infection with an HIV-1 derived vector bearing a CMV-GFP expression cassette and competent for reverse transcription and integration. The extent of infection was determined by flow cytometry 3 days post infection. Averages and SEM of 6 independent experiments are shown here. *statistically significant differences following a Student t test (p≤0.05).
(TIF)

S5 Fig Behavior of Gag and of the E45A capsid mutant on cell entry and on viral infectivity. NL4-3 envelope pseudotyped HIV-1 virions bearing either a single point mutation in CA that leads to the formation of hyperstable viral cores (E45A) or devoid of the entire Pro-Pol (ie Gag only) were produced by DNA transfection of HEK293T cells with plasmids coding the above-mentioned structural proteins and a mini-viral genome competent for reverse transcription and integration and bearing a CMV-GFP expression cassette. Upon purification by ultracentrifugation, virions were normalized by protein content and used to challenge SupT1 cells. Cell entry was measured three hours post viral challenge by FACS using intracellular p24 staining (A), while the extent of infection was assessed 3 days later by flow cytometry (B). Given the lack of viral enzymes, the infectivity of Gag-only virions was not tested here (NT, not tested). The graph presents data obtained with 3 experiments. To better appreciate the entry defect in Gag-only particles, values are presented in log scale. ns; non-significant; *statistically significant difference; Student t test (p≤0.05). The effects of high doses of PF74 on the E45A mutant were further tested following the same experimental scheme described before (C).
(TIF)

S6 Fig Effects of IFNα treatment on the infection of HeLaP4 cells and MDDCs. Dual reporter HIV-1 virions incorporating EU-repRNA and pRRL-GFP, a reverse transcription-integration competent viral genome were produced with the appropriate envelope (derived from NL4-3 or JR-FL) and used to challenge either HeLaP4 cells or MDDCs that had been previously treated for 24 hours with the indicated concentration of IFNα. Flow cytometry analysis was carried out 3 days afterwards. The graph presents data obtained with 4 to 8 independent experiments and, in the case of MDDCs, cells of different donors. *statistically significant difference; Student t test (p≤0.05).
(TIF)
