# Supplementary figures and images for: A Novel Entry/Uncoating Assay Reveals the Presence of at Least Two Species of Viral Capsids During Synchronized HIV-1 Infection
Source: PLoS Pathog. 2016 Sep 30;12(9):e1005897. doi: 10.1371/journal.ppat.1005897 (PMC5045187; doi:10.1371/journal.ppat.1005897)

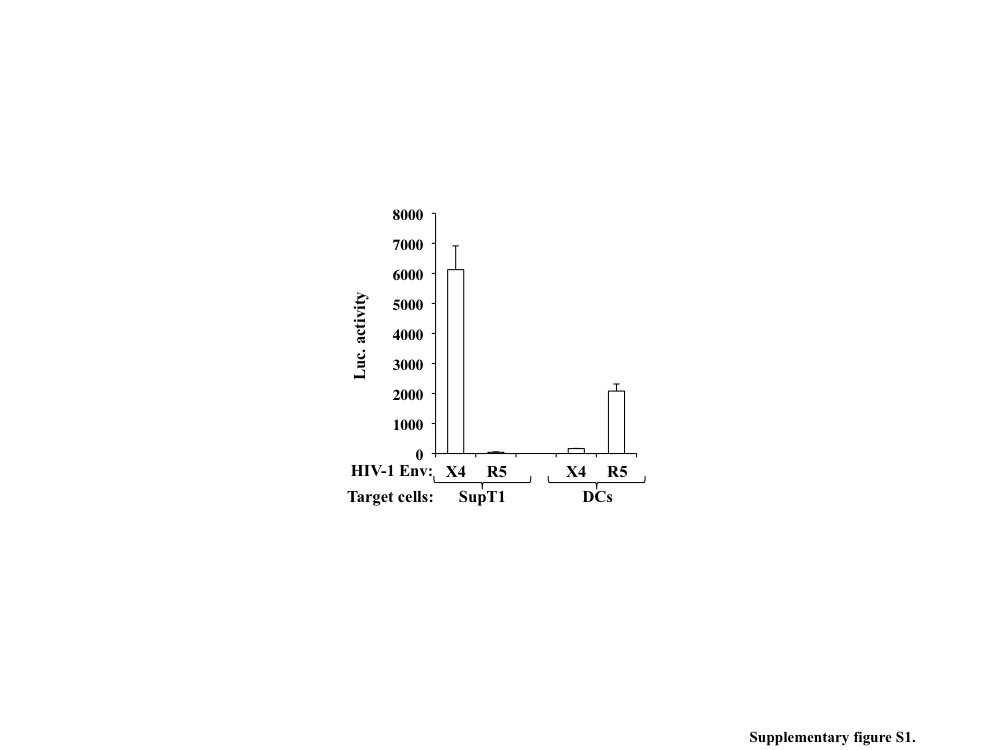

Supplement: S1 Fig — The data in Fig 3A is presented after normalization to controls to allow the direct comparison between different readouts. The graph above presents instead the straight luciferase values obtained following EURT assay, using the same amount of virion particles on the above-mentioned cell types. (TIF) [file ppat.1005897.s001.tif]

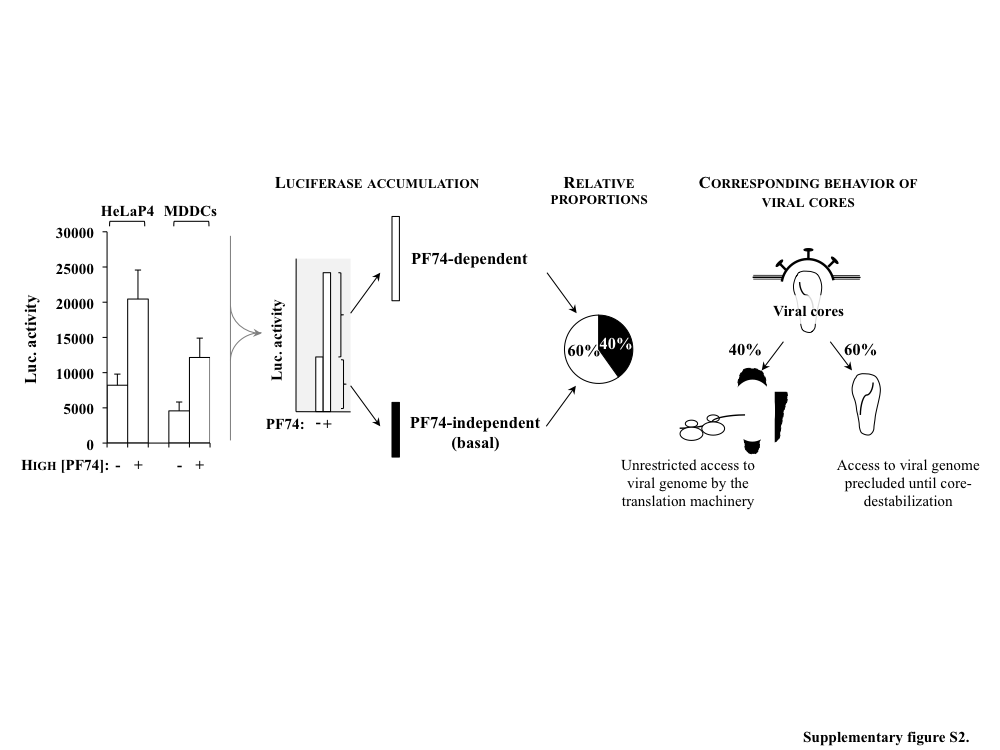

Supplement: S2 Fig — (Left) Non-normalized data related to the graphs of Fig 4C and 4D. (Right) The luciferase activity measured in the presence of PF74 (at 23 μM = 10 μg/mL) includes also the one that can be obtained in the absence of this compound. As such, the relative proportions of PF74-dependent and -independent luciferase accumulation can be measured by subtraction of the basal activity to the overall activity measured during EURT in the presence of PF74. The expected behavior of viral cores with respect to their accession to the translation machinery is also schematically presented. (TIF) [file ppat.1005897.s002.tif]

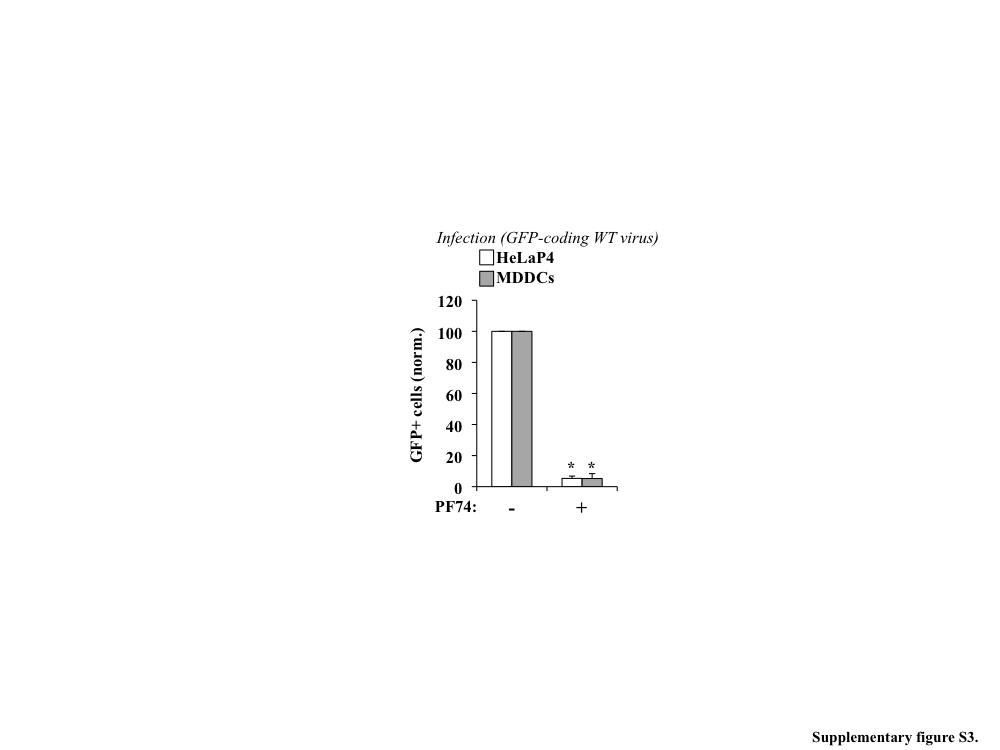

Supplement: S3 Fig — Monocyte-derived dendritic cells were challenged with a GFP-coding JR-FL Env-bearing HIV-1 vector in the presence or absence of PF74 (at 23 μM = 10μg/mL), prior to flow cytometry analysis 3 days after infection. HeLaP4 were instead challenged with NL4-3 envelope-pseudotyped viral particles. The graph presents data obtained from 6 independent experiments. *statistically significant difference following a Student t test (p≤0.05). (TIF) [file ppat.1005897.s003.tif]

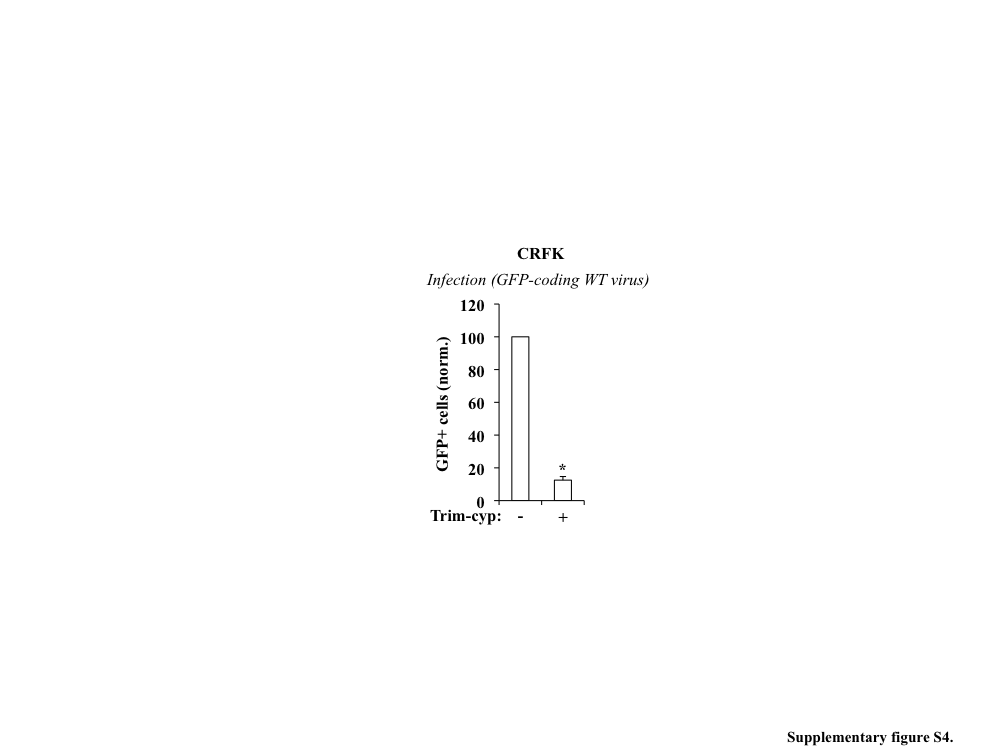

Supplement: S4 Fig — Feline CRFK cells were stably transduced with an MLV-based retroviral vector expressing or not the Owl Monkey Trim5-Cyp fusion protein. Upon puromycin selection, cells were transiently transfected with DNAs coding the HIV-1 receptor/co-receptor CD4 and CXCR4, prior to single round infection with an HIV-1 derived vector bearing a CMV-GFP expression cassette and competent for reverse transcription and integration. The extent of infection was determined by flow cytometry 3 days post infection. Averages and SEM of 6 independent experiments are shown here. *statistically significant differences following a Student t test (p≤0.05). (TIF) [file ppat.1005897.s004.tif]

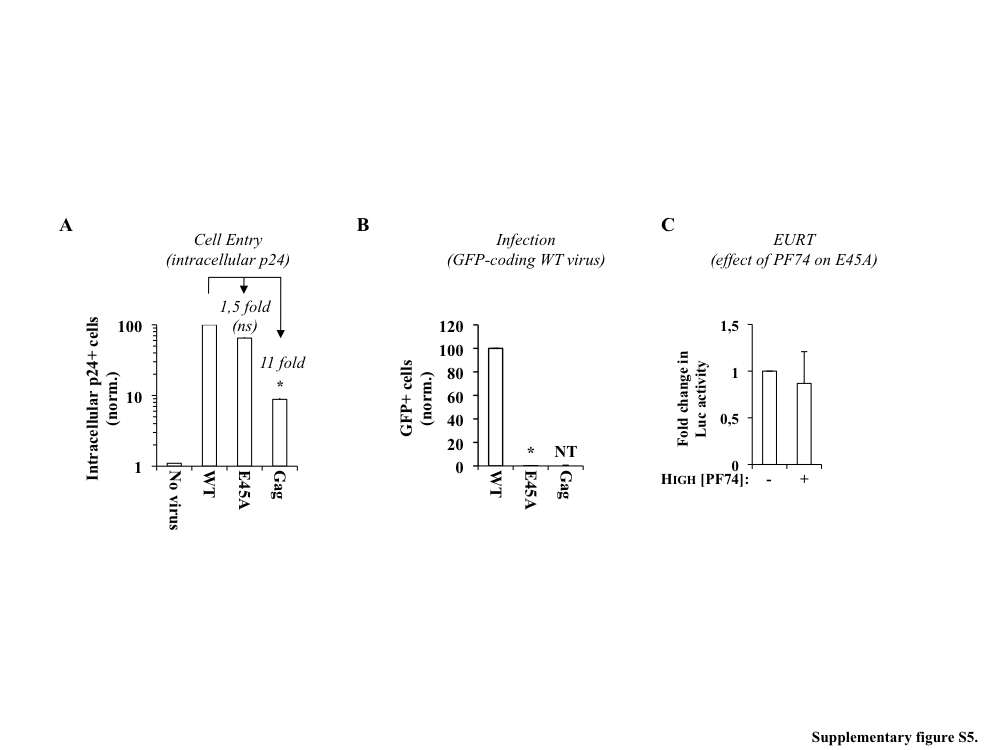

Supplement: S5 Fig — NL4-3 envelope pseudotyped HIV-1 virions bearing either a single point mutation in CA that leads to the formation of hyperstable viral cores (E45A) or devoid of the entire Pro-Pol (ie Gag only) were produced by DNA transfection of HEK293T cells with plasmids coding the above-mentioned structural proteins and a mini-viral genome competent for reverse transcription and integration and bearing a CMV-GFP expression cassette. Upon purification by ultracentrifugation, virions were normalized by protein content and used to challenge SupT1 cells. Cell entry was measured three hours post viral challenge by FACS using intracellular p24 staining (A), while the extent of infection was assessed 3 days later by flow cytometry (B). Given the lack of viral enzymes, the infectivity of Gag-only virions was not tested here (NT, not tested). The graph presents data obtained with 3 experiments. To better appreciate the entry defect in Gag-only particles, values are presented in log scale. ns; non-significant; *statistically significant difference; Student t test (p≤0.05). The effects of high doses of PF74 on the E45A mutant were further tested following the same experimental scheme described before (C). (TIF) [file ppat.1005897.s005.tif]

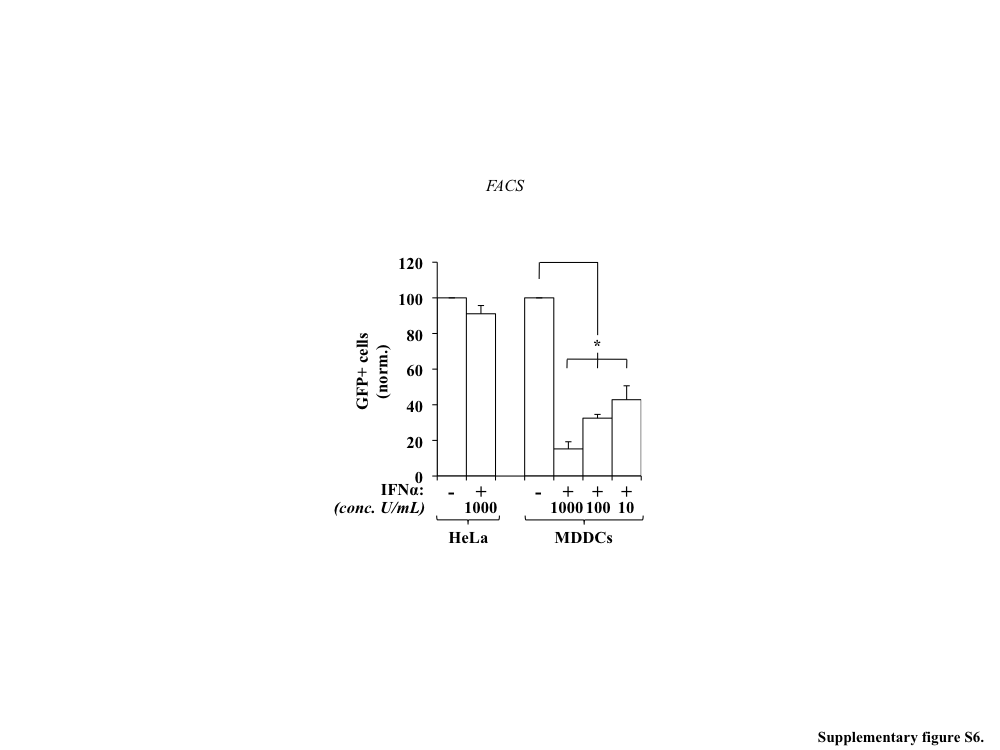

Supplement: S6 Fig — Dual reporter HIV-1 virions incorporating EU-repRNA and pRRL-GFP, a reverse transcription-integration competent viral genome were produced with the appropriate envelope (derived from NL4-3 or JR-FL) and used to challenge either HeLaP4 cells or MDDCs that had been previously treated for 24 hours with the indicated concentration of IFNα. Flow cytometry analysis was carried out 3 days afterwards. The graph presents data obtained with 4 to 8 independent experiments and, in the case of MDDCs, cells of different donors. *statistically significant difference; Student t test (p≤0.05). (TIF) [file ppat.1005897.s006.tif]
